# Differentiation therapy using low‐dose venetoclax in a variant acute promyelocytic leukaemia carrying *ZBTB16‐RARA*

**DOI:** 10.1111/bjh.18476

**Published:** 2022-09-28

**Authors:** He Li, Xinrong Xiang, Hong Ding, Jiang Yu, Juan Xu, Ying Yuan, Yu Wu

**Affiliations:** ^1^ Department of Hematology and Hematology Research Institute West China Hospital, Sichuan University Chengdu People's Republic of China; ^2^ State Key Laboratory of Biotherapy and Cancer Center West China Hospital, Sichuan University Chengdu People's Republic of China; ^3^ Department of Laboratory Medicine West China Hospital, Sichuan University Chengdu People's Republic of China; ^4^ Department of Hematology Guangyuan Central Hospital Sichuan People's Republic of China

**Keywords:** apoptosis, differentiation, venetoclax, *ZBTB16‐RARA*

Acute promyelocyte leukaemia (APL) carrying zinc finger and BTB domain containing 16‐retinoic acid receptor alpha (*ZBTB16‐RARA*) is the most frequently reported variant APL (vAPL) featured with t(11;17)(q23;q21). These vAPLs have dismal prognosis because of induction failure or relapse after chemotherapy.^1^ In vitro and in vivo studies have demonstrated that vAPL carrying *ZBTB16‐RARA* is insensitive to all‐*trans* retinoic acid (ATRA) or arsenic trioxide (ATO) because *ZBTB16‐RARA* lacks the ATO‐binding site and recruits more repressors to suppress RARA targets, differentiation, and apoptosis.^2^ *ZBTB16‐RARA* also confers some ATRA resistance by inducing promoter hypomethylation.^3^

Venetoclax, a BH3 domain targeted BCL2 apoptosis regulator (BCL2) inhibitor, is qualified as a front‐line treatment for older or unfit patients with acute myeloid leukaemia (AML).^4^ Small‐scale studies have provided clinical and molecular evidence on venetoclax treatment in resistant classical APL (PML‐A216V/T‐mutated APL) or vAPL on a default dosage of 400 mg.^5^ Herein, we describe a frail older male patient diagnosed with vAPL carrying *ZBTB16‐RARA* who responded to 100 mg venetoclax monotherapy, suggesting that venetoclax could be a potential induction treatment in vAPL carrying *ZBTB16‐RARA*.

An 82‐year‐old man with a history of chronic obstructive pulmonary disease was transferred to our hospital for intermittent fever, cough, and blood‐tinged sputum in August 2021. Laboratory evaluation revealed a haemoglobin level of 64 g/l (normal range: 120–170 × 10^9^/l), platelet count of 52 × 10^9^/l (normal range: 100–300 × 10^9^/l), white blood cell count of 2.2 × 10^9^/l (normal range: 3.5–9.5 × 10^9^/l), and fibrinogen level of 0.66 g/l (normal range: 2.0–4.0 g/l). Bone marrow examination showed hypercellularity with 81% promyelocytes. Flow cytometry, chromosome karyotyping, and molecular test confirmed the diagnosis of vAPL carrying *ZBTB16‐RARA* with dissemminated intravascular coagulopathy (DIC). Given the poor performance status (Eastern Cooperative Oncology Group Performance Status score 4), compromised cardiopulmonary function, and concurrent pulmonary infection, a combination of ATRA (20 mg twice daily) and ATO was kept due to hypofibrinogenemia correction 3 days after dual‐drug induction, although ZBTB16‐RARA fusion is insensitive to ATRA and ATO. However, 9%–20% of promyelocytic leukaemia cells persistently existed in the peripheral blood, and red blood cell and platelet transfusion dependence was evident after 4 weeks of the dual induction therapy. Considering the patient's age and unfitness, the patient was administered venetoclax (100 mg once a day) as re‐induction therapy. No significant differentiation syndrome or tumour lysis syndrome was noticed. The platelet count improved from day 7 and returned to normal levels after 30 days of venetoclax administration (Figure 1A). Agranulocytosis was observed from day 10 to day 21 and recovered thereafter. Bone marrow aspiration after 30 days of venetoclax administration demonstrated a sharp decrease in promyelocytes to 27% with prominent differentiation in myeloid lineage. Venetoclax was thus continued for another 28 days. During the venetoclax monotherapy, the trough and peak drug levels were 589 and 753 ng/ml respectively, which is lower than the reported venetoclax drug level in AML trials. The bone marrow examination after 60 days showed complete remission, with no minimal residual disease in the flow cytometry. The copy number of *ZBTB16‐RARA* fusion gene decreased by three logs from 52.4% on day 30 to 0.94% on day 60 after venetoclax administration. The patient was then discharged from hospital and continued venetoclax‐based chemotherapy.

**FIGURE 1:**
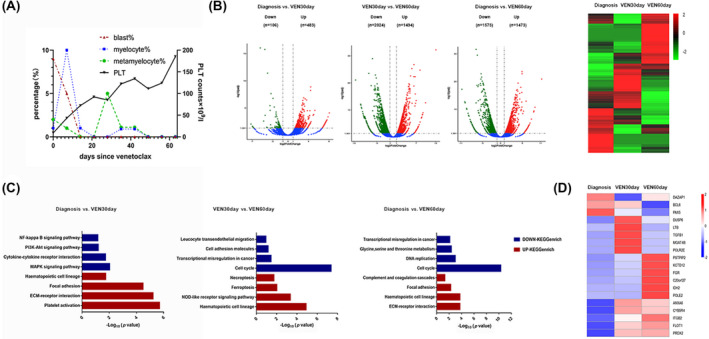
Transcriptional signature of a patient with ZBTB16‐RARA vAPL after venetoclax monotherapy. (A) Peripheral blood blasts, platelets, myelocytes, and percentage during venetoclax monotheraphy. (B) Volcano plot and cluster heat map of DEGs. Up‐ and downregulated DEGs are shown in red and green respectively. (C) KEGG pathways were analysed using ClusterProfiler. The bar chart represents the significance of the gene enrichment for the KEGG pathway. (D) Heat map of the 19 reported ZBTB16‐RARA target genes. Up‐ and downregulated genes are shown in red and blue respectively. DEGs, differentially expressed genes; ECM, extracellular matrix; KEGG, Kyoto Encyclopedia of Genes and Genomes; PLT, platelets; vAPL, variant acute promyelocyte leukaemia; VEN(30day)(60day), (30 days) (60 days) after venetoclax treatment; ZBTB16‐RARA, zinc finger and BTB domain containing 16‐retinoic acid receptor alpha

Bone marrow mononucleated cells on diagnosis, 30 days (VEN30day) and 60 days (VEN60day) after venetoclax treatment were processed and sequenced by Novogene on a HiSeq device (Illumina). RNA‐sequencing analysis was performed on the bone marrow samples at these time‐points to understand the molecular underpinnings of venetoclax monotherapy. Volcano plot and cluster heat map (Figure 1B) displayed the overall changes in differentially expressed gene (DEG) expression. Furthermore, Kyoto Encyclopedia of Genes and Genomes (KEGG) analysis showed that the downregulated genes were significantly enriched in leukaemogenesis pathways, including classic cancer pathways (nuclear factor κ‐light‐chain‐enhancer of activated B cells [NF‐κB], phosphatidylinositol 3‐kinase and protein kinase B [PI3K/Akt], and mitogen‐activated protein kinase [MAPK] pathways), transcriptional dysregulation in cancer, amino acid metabolism, and cell cycle, consistent with previously reported changes during APL leukaemogenesis.^6^ Upregulated genes showed enrichment mainly in the haematopoietic cell lineage, extracellular matrix–receptor interaction, focal adhesion, and different cell death pathways, such as necroptosis, ferroptosis, and nucleotide binding oligomerisation domain (NOD)‐like receptor signalling pathway. These findings are similar to the transcriptome results of the reported vAPLs.^7^ Meanwhile, platelet activation and downregulation of cell adhesion molecules also suggest that our treatment is effective for the DIC phenotype in APL (Figure 1C). Regarding changes in the *ZBTB16‐RARA* pathway of target genes, the 16 upregulated genes and three downregulated genes after venetoclax monotherapy were all reported in the literature (Figure 1D).^8^

Consistent with the upregulation of haematopoietic cell lineage in KEGG and results of noticeable differentiation in the bone marrow examination (Figure 2A), gene set enrichment analysis (GSEA) verifies the differentiation induction of venetoclax, particularly during the second phase of venetoclax treatment with significance (*p* = 0.0389) (Figure 2B). We also focused on the effects of venetoclax on apoptosis. GSEA results showed that specific gene sets related to myeloid cell apoptotic process positively enriched in VEN60day, showing significance in the second phase of venetoclax treatment (*p* = 0.0176) (Figure 2C). Besides, the relative expression of several typical genes related to myeloid cell differentiation (*RARα*, *RARβ*, CCAAT enhancer binding protein alpha [*CEBPA*], CEBP epsilon [*CEBPE*], RUNX family transcription factor 1 [*RUNX1*], colony‐stimulating factor 1 [*CSF1*], KIT proto‐oncogene, receptor tyrosine kinase [*KIT*], etc.) were upregulated significantly after 30 or 60 days of venetoclax administration (Figure 2D). During venetoclax treatment, the levels of anti‐apoptotic proteins BCL2, MCL‐1, BCL‐XL, and BFL1 decreased. Meanwhile, the expression of activator BH3, sensitiser BH3, and pro‐apoptotic effector increased (Figure 2E).

**FIGURE 2:**
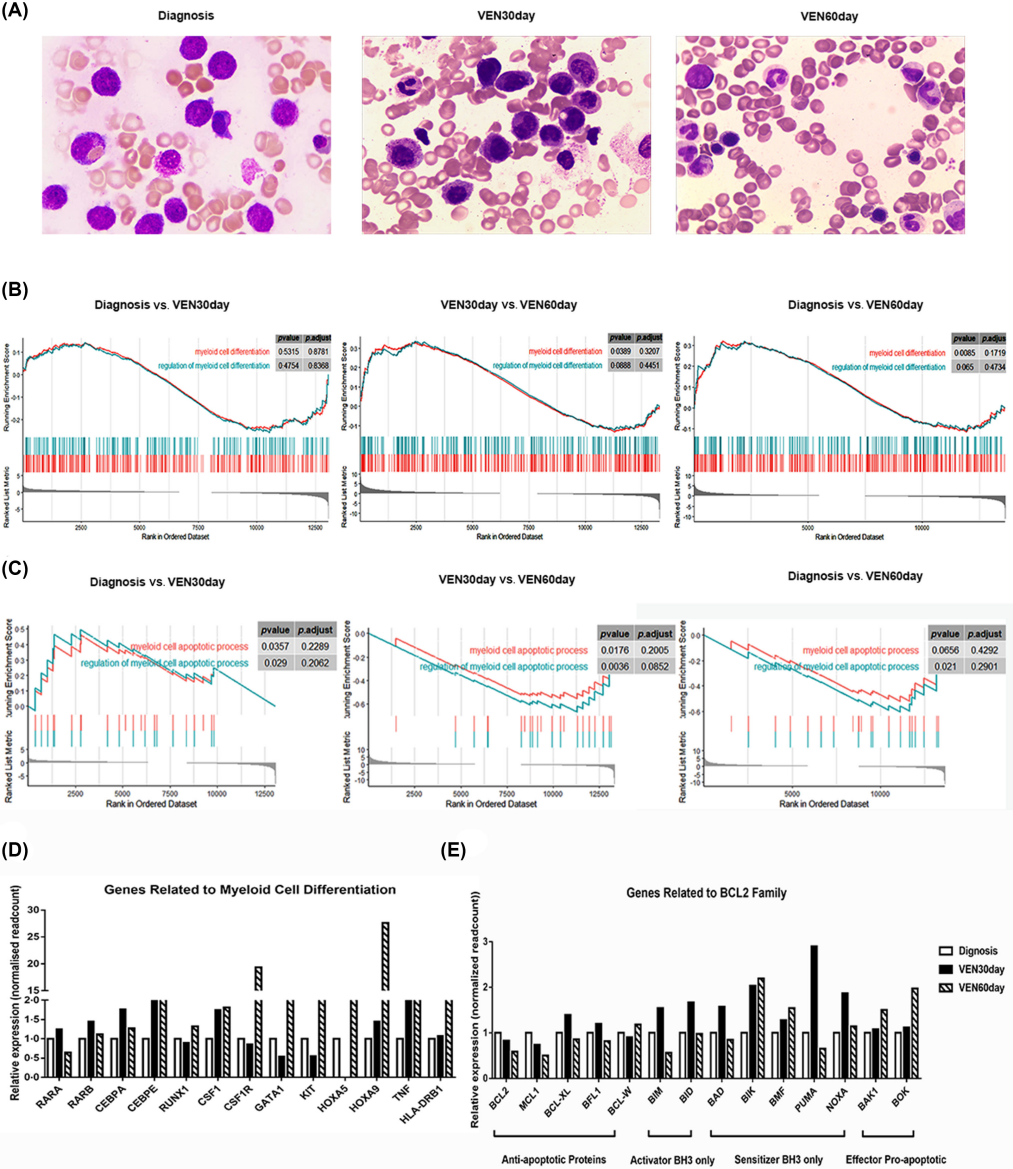
Low‐dose venetoclax induced differentiation and apoptosis in ZBTB16‐RARA. (A) Bone marrow morphology at the time of diagnosis, 30 days of venetoclax and 60 days of venetoclax. (B) GSEA was performed for myeloid cell differentiation. (C) GSEA was performed in myeloid cell apoptotic process. (D) Relative expression of several typical genes related to myeloid cell differentiation after venetoclax treatment. (E) Relative expression of BCL2 family genes after venetoclax treatment. BCL2, BCL2 apoptosis regulator; GSEA, gene set enrichment analysis; ZBTB16‐RARA, zinc finger and BTB domain containing 16‐retinoic acid receptor alpha. VEN(30day)(60day), (30 days) (60 days) after venetoclax treatment

The backbone of conventional induction regimen for variant APL consists of ATRA and AML‐like induction, followed by consolidation therapy with anthracycline‐based regimen or gemtuzumab ozogamicin. The complete remission rate varies between 53.3% and 73%, with a relapse rate of 43%–62.5%. The 3‐year overall survival was 26.7% in contrast with >90% in patients with classical APL.^1^, ^9^, ^10^ This is the first study to report on the efficacy and possible mechanism of low‐dose venetoclax as a differentiation induction therapy in patients with vAPL carrying *ZBTB16‐RARA*. Further in vitro experiments and venetoclax‐based clinical trials are needed to validate this hypothesis.

## AUTHOR CONTRIBUTIONS

Yu Wu, He Li, Juan Xu, Hong Ding, and Ying Yuan participated in the clinical diagnosis and treatment. Jiang Yu completed the morphological analysis of bone marrow. He Li and Xinrong Xiang analysed data and drafted the manuscript.

## CONFLICT OF INTEREST

The authors declare no competing financial interests.

## ETHICS APPROVAL

The study was approved by the West China Hospital of Sichuan University Biomedical Research Ethics Committee (#2019–114).

## PATIENT CONSENT STATEMENT

Written informed consent was obtained from patient.

## Data Availability

For original data, please contact wu_yu@scu.edu.cn.
